# Efficacy of Fu's Subcutaneous Needling on Myofascial Trigger Points for Lateral Epicondylalgia: A Randomized Control Trial

**DOI:** 10.1155/2022/5951327

**Published:** 2022-03-14

**Authors:** Ching-Hsuan Huang, Chun-Yen Lin, Mao-Feng Sun, Zhonghua Fu, Li-Wei Chou

**Affiliations:** ^1^Department of Chinese Traumatology Medicine, China Medical University Hospital, 404332 Taichung, Taiwan; ^2^School of Chinese Medicine, College of Chinese Medicine, China Medical University, 406040 Taichung, Taiwan; ^3^Lin Clinic, 404332 Taichung, Taiwan; ^4^Graduate Institute of Acupuncture Science, College of Chinese Medicine, China Medical University, 406040 Taichung, Taiwan; ^5^Institute of Fu's Subcutaneous Needling, Beijing University of Chinese Medicine, 100029 Beijing, China; ^6^Department of Physical Medicine and Rehabilitation, China Medical University Hospital, 404332 Taichung, Taiwan; ^7^Department of Physical Therapy and Graduate Institute of Rehabilitation Science, China Medical University, 406040 Taichung, Taiwan; ^8^Department of Physical Medicine and Rehabilitation, Asia University Hospital, Asia University, 413505 Taichung, Taiwan

## Abstract

Lateral epicondylalgia (LE), a common overuse syndrome of the extensor muscle and tendons on the lateral epicondyle, causes persistent severe musculoskeletal pain on the outer part of the elbow. Fu's subcutaneous needling (FSN), a newly invented subtype of acupuncture and dry needling, is a new trend and potential treatment of LE by targeting the myofascial trigger points (MTrPs). However, no scientific evidence is available to support this method. This study aims to evaluate the distal FSN treatment on the LE by measuring pain-related scales, such as visual analog scale (VAS), pressure pain threshold (PPT), muscle tissue hardness (TH), pain-free grip (PFG), and the functional outcome by a patient-rated tennis elbow evaluation (PRTEE) questionnaire study. A total of 60 LE patients were randomly divided into FSN (*n* = 30) and transcutaneous electrical nerve stimulation (TENS, *n* = 30) as the control group. Every subject was treated with three regimens and followed up for 15 days. Results showed that FSN has an immediate effect on VAS, PPT, TH, and PFG. Moreover, sustained effects on pain relief were followed up to 15 days. Pain remission was consistent with long-term PRTEE results. Overall, FSN is a safe and efficient therapy option for LE, significantly improving pain relief and activity difficulty with immediate, short-term, and long-term effectiveness. This trial is registered with ClinicalTrials.gov NCT03605563.

## 1. Introduction

Lateral epicondylalgia (LE), also known as tennis elbow, is one of the musculoskeletal disorders and is the most common cause of elbow pain due to overuse of the extensor muscle and tendons [1]. The prevalence of LE is approximately 1% to 3% of the overall population; however, LE is found in up to 23% of male tennis athletes [2, 3]. The pain and tenderness caused by LE are highly related to repeated and forceful contractions of the wrist and fingers muscles, which is commonly seen in griping and holding continuously for a long time [4]. Lateral elbow tendinopathy is a common condition and is used to assess the severity of LE despite the absence of pathological bone lesion of the elbow in radiographic imaging with large intrasubstance tears identified by musculoskeletal sonography [5]. The thick common extensor tendon, bone spurs, and color Doppler activity of the elbow are often found in LE patients [6].

Although most patients recover within one year, some patients have a long disease course up to two years [7]. Conventional treatments, including local injections with anesthetics [8], steroids [9], and platelet-rich plasma [10], extracorporeal shock waves [11], transcutaneous electrical nerve stimulation (TENS) [12], acupuncture [13, 14], and dry needling [15, 16] are widely used. However, most of these treatments only provide short-term pain relief. The most effective treatment for LE remains to be inconclusive.

Fu's subcutaneous needling (FSN) is a new technique originated from Chinese acupuncture, which utilizes the dual structure trocar and chooses the site away from the lesion, targeting the myofascial trigger points (MTrPs) in the taut band with needling insertion and sway movement in the superficial fascia. FSN has the advantages of few needle requirements and no need to penetrate to the deep layers of the derma and muscle generally. FSN has an immediate and long-lasting effect on pain control of musculoskeletal diseases through the swaying and reperfusion approach [17, 18]. For example, FSN has also been reported as an effective treatment for low back pain [19, 20]. MTrP in the extensor carpi radialis brevis and extensor digitorum communis muscle can reproduce the pain of LE [21]. As a new technique of acupuncture and dry needling, FSN has been recently applied to treat the chronic recurrent LE clinically without adverse side effects. However, the scientific evaluation of FSN on short- and long-term effectiveness is still lacking.

This study utilized subjective and objective outcome measurements, such as visual analog scale (VAS), pressure pain threshold (PPT), muscle tissue hardness (TH), pain-free grip (PFG), and patient-rated tennis elbow evaluation (PRTEE) questionnaire, to assess the efficacy of the FSN treatment compared with TENS on the LE patients.

## 2. Materials and Methods

### 2.1. Study Subjects

A total of 60 participants were recruited in accordance with a randomized, controlled, open-label experiment. This study was approved by the Institutional Review Board of the China Medical University Hospital. All patients gave their written informed consent to participate in this study, and the research was conducted in accordance with the principles of the Declaration of Helsinki.

The inclusion criteria are as follows: (1) age over 20; (2) diagnosed with LE for more than one month and subjective VAS >5; and (3) local tenderness on lateral epicondyle with exacerbating pain under isometric resistant test for supination of the forearm. Meanwhile, the exclusion criteria include the following: (1) patients who previously received operations for the neck, upper back, or four limbs; (2) currently undergoing different therapies for LE; and (3) equipped with a pacemaker, diagnosed with epilepsy, or other conditions, such as skin injury, contributing to the inapplicability of electric patch.

**Figure 1:**
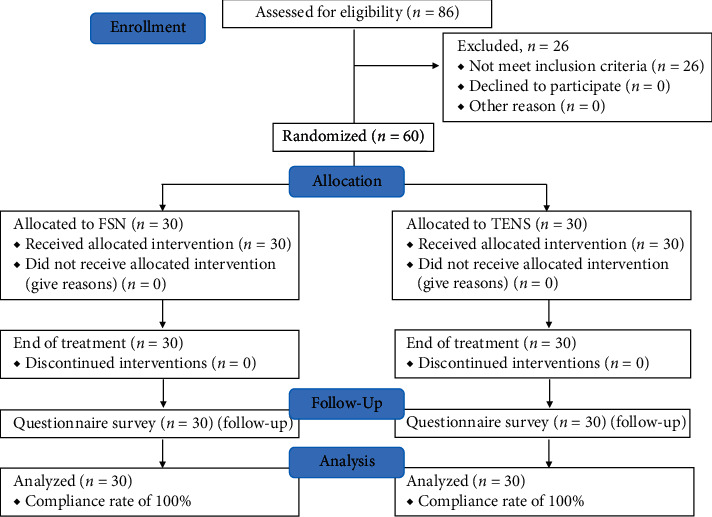
Study flow chart.

Every subject was randomly allocated into two groups: an experimental group who will undergo FSN treatment, and a placebo group who will undergo TENS treatment (Figure 1). The entire course lasts for two weeks. A total of three treatment sessions will be performed in the first week, with assessment before each treatment session and immediately after treatment as well as the 1st and 2nd week for follow-up. All the treatments were conducted by the same acupuncturist who worked in the medical center in Taiwan for more than five years.

### 2.2. Fu's Subcutaneous Needling (FSN)

**Figure 2:**
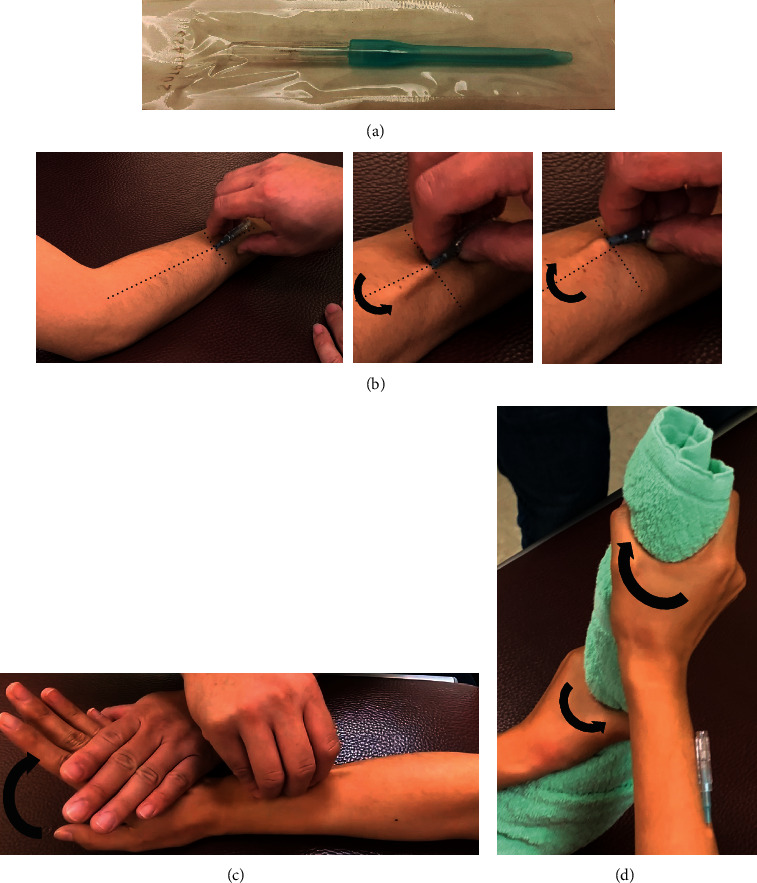
Illustration of the operations of the Fu's subcutaneous needling. (a) Fu's subcutaneous needle. (b) Puncture site away from the elbow, and starting a swaying movement. (c) Wrist extension movement. (d) Wringing a towel.

The forearm of the experimental group relied on using a disposable Fu's subcutaneous needle (Nanjing FSN Medical Co., Ltd., Jiangsu, China) to treat the radial aspect of the forearm extensor muscle (Figure 2(a)). The puncture site was on the midpoint of the extensor muscle of the forearm, in which the tip of the needle was pointed toward the lateral epicondyle (Figure 2(b)) and inserted into the subcutaneous layer with the entire needle body (Video S1).

Receding the core needle and then fixing the protuberance of the soft tube seat in the slot of the core seat prevent the exposure of the needle tip outside followed by starting a swaying movement. The tip of the needle should be maintained at the same horizontal level in swaying by using the thumb and the middle finger holding the core base, and the index and ring fingers are separated on the left and right side of the middle finger to sway in a seesaw-like sector one after the other (Figure 2(b) and Video S2). The time and frequency of swaying are 50 times within 30 s. The subjects would be asked to perform wrist extension movement with resistance for 10 s after swaying and then rest for another 10 s (Figure 2(c)). The cycle is repeated up to three times for 1 min. The subjects would then be asked to simulate actions of wringing a towel (forearm supination and pronation) for 10 s and rest for 10 s, also repeating the cycle up to three times for 1 min (Figure 2(d)). The needle is then removed after completing the two movements, namely the “reperfusion approach,” with the subcutaneous embedding of FSN.

### 2.3. Transcutaneous Electrical Nerve Stimulation

**Figure 3:**
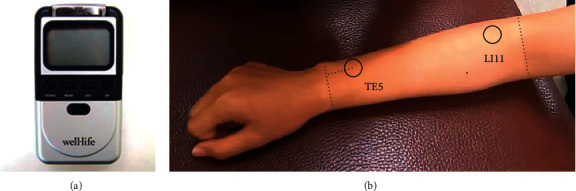
Illustration of the transcutaneous electrical nerve stimulations (a) and electrodes attached sites on TE5 and LI11 (b).

The forearm of the placebo subjects was treated with a transcutaneous electrical nerve stimulator (Well-Life Healthcare Limited, Taiwan, Figure 3(a)), with 2 electrodes attached to acupoints, namely TE5 (*Waiguan*) and LI11 (*Quchi*), according to the guidance of the WHO (Figure 3(b)), the two most commonly used acupoints for LE treatment by acupuncturists [22]. The treatment parameters were set to a pulse of width 200 *μ*s, a frequency of 200 Hz, and a continuous wave for 20 min.

### 2.4. Outcome Measurements

1. VAS is a subjective pain intensity questionnaire for pain severity [23]. Patients evaluated the score of pain severity from no pain (score zero) to intolerable pain (score ten) before and after treatment.
2. PPT is a semiobjective quantification tool. Pressure algometry is used to measure the PPT on MTrPs by following Fischer's standard method [24, 25].
3. The PFG test is commonly performed using a grip dynamometer to measure the amount of force of grasp (kg) generated by LE patients upon the onset of pain [26]. The test started with a normal arm, wherein subjects holed the grip dynamometer (Jamar® Plus + Digital Hand Dynamometer, Performance Health, IL US) with a relax and extension status and slowly began griping until the onset of pain. No pain of the test result was recorded as the maximum grip strength. The testing was repeated three times with 1 min rest intervals. The same procedure was performed on the affected arm. The average of PFG was then calculated and recorded.
4. TH assessment of the forearm muscle revealed the capability of muscles against deformation during activities. [27] Patients with LE often suffered from the stiffness of the extensor muscle near the elbow joint, and their activities are limited. TH is determined by using a myometer (OE-220, purchased from ITO CO., Ltd., Tokyo, Japan), a noninvasive and objective electronic device, thus providing an accurate diagnosis for clinicians to determine worse situations involving the muscle [28].
5. The PRTEE questionnaire was regularly used to measure perceived pain and disability [29]. A total of 15 self-reported questionnaires involved in pain, usual activities, and specific activities scaled from 0 (no pain or difficulty) to 10 (worst ever or unable to perform). The subjects were asked to answer the questionnaire before the treatment on day 1 and were then followed up on days 8 and 15 post treatment.

### 2.5. Statistics

Statistically significant differences (*P* < 0.05) among the results were revealed by using Statistical Package for Social Science (SPSS 18.0) for Windows. Data were expressed as mean ± standard deviation. The analysis of baseline characteristics of age, sex, VAS, PTT, TH, PFG, and PRTEE was conducted by analysis of variance (ANOVA). The within-group analysis of all variables was conducted by paired sample *t*-test for inferential statistics, while the between-group analysis of the variables was performed by independent two-sample *t*-test.

## 3. Results and Discussion

### 3.1. Baseline Characteristics of the Subjects of the Two Groups in the Study

All subjects were randomly divided into FSN and TENS groups, and the baseline characteristics are shown in Table 1. The mean age was 47 in both groups. A total of 21 males and 39 females were enrolled and assigned randomly. No significant difference was found between the two groups of age, gender, pre-Tx value of VAS, PPT, TH, PFG, and PRTEE. Thus, this study is a well-randomized prospective investigation for the advanced measurement of effectiveness.

### 3.2. Immediate Effect of FSN and TENS

**Figure 4:**
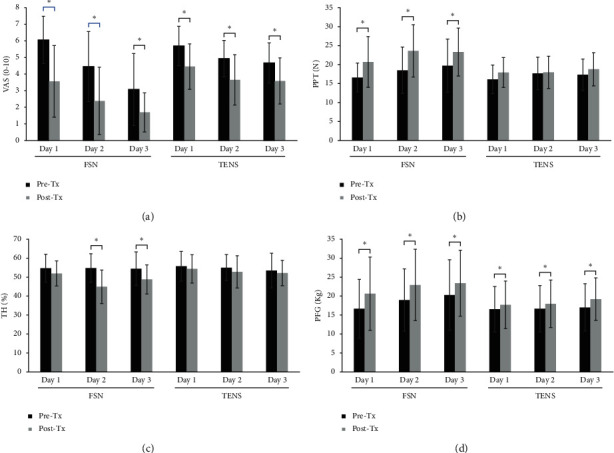
Comparison of the immediate effects of the two groups. The pre- and post-treatment values of VAS (a), PPT (b), TH (c), and PFG (d) were measured in 3 treatment sessions in both groups. ^∗^showed the *P* < 0.05. VAS: visual analog scale, PPT: pressure pain threshold, TH: tissue hardness, PFG: pain-free grip.

The pain-related scales, such as VAS, PPT, TH, and PFG, are compared in pre-and post-treatment to evaluate the immediate effectiveness of FSN (Table 2). VAS (pre-Tx, 6.06 ± 1.43; post-Tx, 3.56 ± 2.16, *P* < 0.01), PPT (pre-Tx, 16.60 ± 3.80; post-Tx, 20.71 ± 6.69, *P* < 0.01), and PFG (pre-Tx, 16.66 ± 7.76; post-Tx, 20.62 ± 9.67, *P* < 0.01) were significantly improved in the FSN group in day 1 (Figures 4(a)–4(d)). Meanwhile, only VAS (pre-Tx, 5.70 ± 1.19; post-Tx, 4.45 ± 1.37, *P* < 0.01) and PFG (pre-Tx, 16.52 ± 5.99; post-Tx, 17.68 ± 6.28, *P*=0.01) were significantly improved for TENS (Figures 4(a) and 4(d)). VAS, PPT, TH, and PFG were significantly improved in the FSN group on days 2 and 3 (*P* < 0.01 in all tests, Figures 4(a)–4(d)). Only VAS (days 2 and 3) and PFG (days 2 and 3) were significantly improved in the TENS group (Figures 4(a) and 4(d)). These results indicated that FSN had an instant pain relief effect for LE with a decrease in VAS and TH and an increase in PPT and PFG compared with TENS.

### 3.3. Short- and Long-Term Effects of FSN and TENS

**Figure 5:**
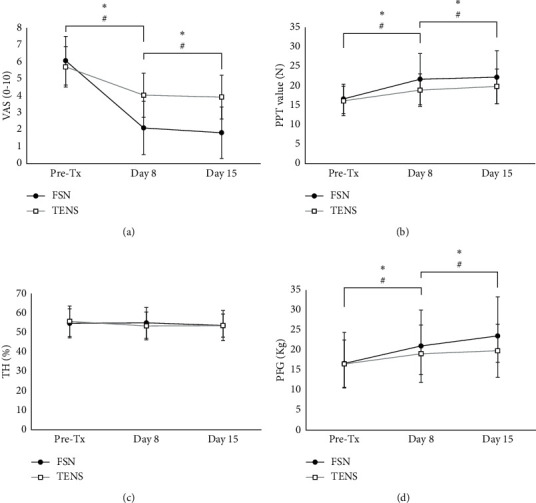
Comparison of the short-term and long-term effects of the two groups. The value of VAS (a), PPT (b), TH (c), and PFG (d) was measured in day 1 pretreatment and followed up to day 8 and day 15 in both groups. ^∗^ and ^#^showed the *P* < 0.05 in FSN or TENS, respectively. VAS: visual analog scale, PPT: pressure pain threshold, TH: tissue hardness, PFG: pain-free grip, FSN: Fu's subcutaneous needling, TENS: transcutaneous electrical nerve stimulations.

Patients were followed up on days 8 and 15 after treatment to evaluate the short- and long-term effects and investigate whether pain relief is a sustained effect by FSN (Table 3). VAS in the FSN group was scored from 6.06 ± 1.43 in day 1 pretreatment to 2.10 ± 1.57 in day 8 (*P* < 0.01) and 1.82 ± 1.52 in day 15 (*P* < 0.01, Figure 5(a)). Meanwhile, VAS in the TENS group also demonstrated significant improvements on days 8 and 15 (*P* < 0.01) compared with day 1 pretreatment. Except for TH, PPT and PFG revealed significant improvements on days 8 and 15 (*P* < 0.01) compared with day 1 pretreatment in both groups (Figures 5(b)–5(d)).

To further understand the superiority of FSN to TENS on LE treatment. The improved VAS of patients in FSN on days 8 and 15 was significantly higher than that in TENS (day 8, 3.96 ± 1.46 in FSN compared with 1.67 ± 1.21 in TENS, *P* < 0.01; day 15, 4.24 ± 1.45 in FSN compared with 1.88 ± 1.23 in TENS, *P* < 0.01, Table 4). No significant improvement was noted in PPT on day 8 or 15 of FSN or TENS. The improved value of PFG in FSN was significantly higher than that in TENS (*P* < 0.01). These results indicated the sustained effect of FSN on pain relief.

### 3.4. Improving the Perceived Pain and Disability by FSN

**Figure 6:**
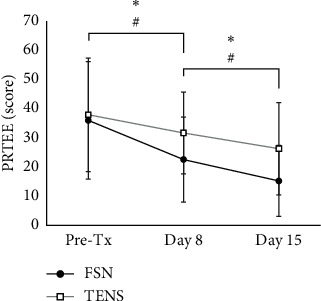
Comparison of the improvement effect of the two groups. The score of the PRTEE questionnaire was evaluated before the treatment on day 1, followed up on day 8 and day 15 post treatment in both groups. ^∗^ and ^#^showed the *P* < 0.05 in FSN or TENS, respectively. PRTEE: Patient-Rated Tennis Elbow Evaluation Questionnaire; FSN: Fu's subcutaneous needling; TENS: transcutaneous electrical nerve stimulations.

The PRTEE questionnaire was utilized for assessment to understand FSN for achieving LE-induced pain remission and disability improvement (Table 5). A significant decrease in score is notably observed from 35.97 ± 20.13 in day 1 pretreatment to 22.57 ± 14.57 in day 8 (*P* < 0.01) and to 15.23 ± 12.16 in day 15 (*P* < 0.01, Figure 6) by FSN. Moreover, a significant decrease in the score is observed from 37.90 ± 19.45 on day 1 to 31.67 ± 14.00 on day 8 (total of 6.23 difference, *P*=0.01) and to 26.27 ± 15.77 on day 15 (total of 11.63 difference, *P* < 0.01, Figure 6) by TENS. Overall, both treatment groups were effective. However, the improvement of FSN is more than TENS in days 8 and 15 compared with day 1 pretreatment (improvement score followed up to day 8, 13.40 ± 15.83 in FSN and 6.23 ± 16.82 in TENS, *P* < 0.01; day 15, 20.74 ± 14.56 in FSN and 11.63 ± 16.28 in TENS, *P* < 0.01, Table 6). These results demonstrated that FSN not only has a sustained pain relief effect but also improved the disability in LE patients.

## 4. Discussion

Nonsurgical therapies are the leading options for treating LE patients. FSN and TENS are compared in this study by measuring pain-related scales, such as VAS, PPT, PFG, and TH, and the pain relief and improvement on disability after three treatment sessions are estimated. The results indicate that FSN reduced the elbow pain and improved the disability caused by LE. Remarkable progress in immediate remission of VAS, PPT, TH, and PFG within three days is also observed. FSN also had short- and long-term benefits on VAS and PFG compared with TENS. Subjective questionnaire research showed an impressive improvement on LE-specific pain and disability. These results indicated that FSN is an effective treatment for LE.

TENS was used clinically to reduce pain by activating large-diameter afferents to reduce nociceptive input via inhibitory neurotransmitters as gate control theory [30, 31] by the electrical stimulation electrodes attached to the affected sites. This mechanism resulted in immediate pain relief of MTrP-induced pain [32], but no benefit was found for reducing the muscle tissue hardness or recovering the function as observed on PPT and TH in the present study. FSN could reduce muscle tissue hardness and increase the PPT by up to two weeks. These results suggested that FSN has better benefits than TENS on pain relief by improving muscle functions. Unlike TENS treated on TE5 and LI11 near the lateral epicondyle, FSN is performed on the midpoint of the forearm extensors with some distance from the lateral epicondyle, demonstrating a more significant outcome than treating the symptom at the pain site. Thus, the etiology of pain may come from MTrPs, not the proximal sites where the pain occurred [33].

The MTrP-associated muscle, namely “tightened muscle,” is essential for diagnosis and has been the treatment target of FSN because most pathological tightened areas were located in the muscle belly, and these areas were generally felt similar to a sheet or a zone, not a point [33]. Two decades ago, FSN was first discovered in muscle disease research [34]. Parallel or vertical needling has the same effectiveness with swaying movement in superficial fascia as first demonstrated in 2007 [35]. The mechanism of action of conventional acupuncture is hypothesized to be a mechanical coupling between the needle and connective tissue starting from needle insertion and rotation and then transmits the signal to connective tissues via mechanotransduction [36]. Some researchers had attempted to explain the phenomenon of acupuncture, that is, piezoelectric effects [37, 38]. They assumed that the electric current was initially generated by pressing the skin and then transduced to connective tissues by needle insertion and rotation. Several biological organic compounds, such as collagen, have piezoelectric properties associated with their crystalline structure [39, 40]. The liquid semicrystalline state of collagen polymers exhibited piezoelectric polarization [40]. However, demonstrating the mechanism of the piezoelectric effect during acupuncture is difficult. Dr. Lin recently developed an in-glove sensor to monitor piezoelectricity from the acupuncture performer [41]. This device may be used for demonstrating piezoelectricity in acupuncture in the future.

The swaying movement of FSN in a seesaw-like sector could stretch loose connective tissues to modulate homeostasis [42, 43]. Extruding, stretching, and swaying by FSN could change the liquid crystalline nanoarchitectures of loose connective tissues to release bioelectricity following transduction piezoelectricity to reverse piezoelectricity in the lesions. This mechanism could open the ion channel to relieve muscle contracture and ischemia and restore muscle function [44]. The reperfusion approach is another feature of FSN. The LE subjects would be asked to simulate actions of wringing a towel (supination and pronation of the forearm) during the treatment. This action would largely increase the bloodstream and congestion regions to promote tissue recovery. Combining swaying and reperfusion is essential for FSN to relieve pain and disability due to the pathological tightened muscle.

Moreover, the diagnosis of the location of the pathological tightened muscle is crucial. The midpoint of the forearm extensors in this study was punctured with some distance from the lateral epicondyle, which is the common site of pathological tightened muscles. Dr. Simons (2002) revealed the etiological mechanism of taut band induced by MTrPs [45], and the application of needling therapy on the active MTrP via breaking the energy crisis and resetting the MTrP circuits was suggested [46]. Some tensed and shortened sarcomeres could cause the extension of nearby sarcomeres. LE may not arise from the lateral epicondyle. The study of PPT on MTrPs in 550 healthy children with ages ranging from 4 to 11 revealed that PPT on the midpoint of the muscle belly was substantially decreased than lateral epicondyle at the elbow and the muscle-tendon junction after the age of 9 years [47]. Curing a disease should focus on its etiology and finding a treatment strategy rather than the name [48]. Therefore, this study focused on the pathological tightened muscle of LE rather than the lateral epicondyle at the elbow.

The mechanical pain is due to microwound accumulation from an imbalance in global muscle recruitment or local muscle overuse [49]. The muscle tissue involved in unscrewing the cap or wringing a towel is still in an overtense state without movement, which is the target of FSN treatment. Reperfusion action to perform wrist extension movement with resistance and wringing a towel in FSN increased the PFG, indicating that FSN-improved muscle function and pain relief immediately. This effect was also observed in one- and two-week follow-ups. These pieces of evidence support that FSN is a good treatment strategy for LE.

## 5. Conclusions

This study provides evidence to support the effectiveness of FSN as a therapy for LE with immediate and sustained effects to relieve pain. Furthermore, the data from the questionnaire study suggest a significant and positive effect of FSN for LE treatment. Thus, FSN is a good treatment option for LE.

## Figures and Tables

**Table 1:** Baseline characteristics and clinical evaluation indicators of the subjects in the two groups.

Groups	FSN	TENS	*P* value
Number of subjects	30	30	
Age (years)	47.97 ± 11.85	47.10 ± 12.15	0.80
Range of age (years)	26–70	28–70	
Sex			
Male (*n* = 21)	11	10	0.90
Female (*n* = 39)	19	20	0.90
Pre-Tx VAS (score 0–10)	6.06 ± 1.43	5.70 ± 1.19	0.43
Pre-Tx PPT (N)	16.60 ± 3.80	16.11 ± 3.79	0.54
Pre-Tx TH (%)	54.68 ± 7.43	55.70 ± 7.85	0.90
Pre-Tx PFG (Kg)	16.66 ± 7.06	16.52 ± 5.99	0.89
Pre-Tx PRTEE (score)	35.97 ± 20.13	37.90 ± 19.45	0.66

Data were expressed as mean ± SD; *P* value was tested with ANOVA. FSN: Fu's subcutaneous needling; TENS: transcutaneous electrical nerve stimulations; VAS: visual analog scale; PPT: pain pressure threshold; TH: tissue hardness of muscle; PFG: pain-free grip; PRTEE: Patient-Rated Tennis Elbow Evaluation Questionnaire.

**Table 2:** The immediate effects of the two groups.

	FSN			TENS		
	Pre-Tx	Post-Tx	*P* value	Pre-Tx	Post-Tx	*P* value
Day 1						
VAS (1–10)	6.06 ± 1.43	3.56 ± 2.16	< 0.01	5.70 ± 1.19	4.45 ± 1.37	<0.01
PPT (N)	16.60 ± 3.80	20.71 ± 6.69	< 0.01	16.11 ± 3.79	17.92 ± 3.95	0.06
TH (%)	54.68 ± 7.43	51.93 ± 6.65	0.06	55.70 ± 7.85	54.37 ± 7.46	0.11
PFG (Kg)	16.66 ± 7.76	20.62 ± 9.67	< 0.01	16.52 ± 5.99	17.68 ± 6.28	0.01
Day 2						
VAS (1–10)	4.45 ± 2.11	2.38 ± 2.03	< 0.01	4.93 ± 1.10	3.65 ± 1.51	<0.01
PPT (N)	18.52 ± 6.14	23.63 ± 6.90	< 0.01	17.69 ± 4.29	17.98 ± 4.27	0.32
TH (%)	54.81 ± 7.56	44.88 ± 8.81	< 0.01	55.01 ± 6.93	52.77 ± 8.51	0.06
PFG (Kg)	18.96 ± 8.22	22.92 ± 9.43	< 0.01	16.66 ± 6.09	17.96 ± 6.26	0.04
Day 3						
VAS (1–10)	3.07 ± 2.17	1.69 ± 1.18	< 0.01	4.67 ± 1.21	3.58 ± 1.39	<0.01
PPT (N)	19.73 ± 7.02	23.32 ± 6.33	< 0.01	17.33 ± 4.12	18.77 ± 4.38	0.08
TH (%)	54.47 ± 8.81	48.80 ± 7.69	< 0.01	53.47 ± 9.15	52.14 ± 6.67	0.18
PFG (Kg)	20.28 ± 9.31	23.39 ± 8.71	< 0.01	17.00 ± 6.25	19.19 ± 5.60	<0.01

Data were expressed as mean ± SD; *P* value was analyzed by paired *t*-test. FSN: Fu's subcutaneous needling; TENS: transcutaneous electrical nerve stimulations; VAS: visual analog scale; PPT: pain pressure threshold; TH: tissue hardness of muscle; PFG: pain-free grip; PRTEE: Patient-Rated Tennis Elbow Evaluation Questionnaire.

**Table 3:** The short-term and long-term effects of FSN-improved value are compared to those of TENS.

	Pre-Tx in day 1	Day 8	*P* value^*a*^	Day 15	*P* value^*b*^
VAS (0–10)					
FSN	6.06 ± 1.43	2.10 ± 1.57	<0.01	1.82 ± 1.52	<0.01
TENS	5.70 ± 1.19	4.03 ± 1.30	<0.01	3.92 ± 1.29	<0.01
PPT (N)					
FSN	16.60 ± 3.80	21.67 ± 6.60	<0.01	22.18 ± 6.78	<0.01
TENS	16.11 ± 3.79	18.87 ± 4.16	<0.01	19.83 ± 4.44	<0.01
TH (%)					
FSN	54.68 ± 7.43	54.94 ± 7.96	0.45	53.58 ± 7.74	0.27
TENS	55.70 ± 7.85	53.36 ± 7.17	0.10	53.51 ± 5.95	0.09
PFG (Kg)					
FSN	16.66 ± 7.76	21.02 ± 8.96	<0.01	23.54 ± 9.75	<0.01
TENS	16.52 ± 5.99	19.08 ± 7.18	<0.01	19.83 ± 6.63	<0.01

Data were expressed as mean ± SD; *P* value was analyzed by paired *t*-test. ^a^Comparison of the value in pre-Tx and in day 8 of the FSN or TENS group. ^b^Comparison of the value in day 8 and in day 15 of the FSN or TENS group. FSN: Fu's subcutaneous needling; TENS: transcutaneous electrical nerve stimulations; VAS: visual analog scale; PPT: pain pressure threshold; TH: tissue hardness of muscle; PFG: pain-free grip.

**Table 4:** The improvement effects of FSN compared to those of TENS.

	FSN	TENS	*P* value
Day 8			
VAS (0–10)	3.96 ± 1.46	1.67 ± 1.21	<0.01
PPT (N)	5.07 ± 4.45	2.76 ± 3.98	0.05
PFG (Kg)	4.36 ± 8.32	2.56 ± 6.28	0.07
Day 15			
VAS (0–10)	4.24 ± 1.45	1.88 ± 1.23	<0.01
PPT (N)	5.58 ± 4.82	3.72 ± 4.13	0.11
PFG (Kg)	6.68 ± 8.13	3.31 ± 6.35	<0.01

Data were expressed as mean ± SD; *P* value was analyzed by the *t*-test. FSN: Fu's subcutaneous needling; TENS: transcutaneous electrical nerve stimulations; VAS: visual analog scale; PPT: pain pressure threshold; PFG: pain-free grip.

**Table 5:** The effectiveness of FSN and TENS on PRTEE score.

Group	Pre-Tx in day 1	Day 8	*P* value	Day15	*P* value
FSN	35.97 ± 20.13	22.57 ± 14.57	<0.01	15.23 ± 12.16	<0.01
TENS	37.90 ± 19.45	31.67 ± 14.00	0.01	26.27 ± 15.77	<0.01

Data were expressed as mean ± SD; *P* value was analyzed by the *t*-test. FSN: Fu's subcutaneous needling; TENS: transcutaneous electrical nerve stimulations; PRTEE: Patient-Rated Tennis Elbow Evaluation Questionnaire.

**Table 6:** The improvement effect of PRTEE in FSN compared to that in TENS.

	FSN	TENS	*P* value
Day 8	13.40 ± 15.83	6.23 ± 16.82	<0.01^*∗*^
Day 15	20.74 ± 14.56	11.63 ± 16.28	<0.01^*∗*^

Data were expressed as mean ± SD; *P* value was analyzed by the *t*-test. FSN: Fu's subcutaneous needling; TENS: transcutaneous electrical nerve stimulations; PRTEE: Patient-Rated Tennis Elbow Evaluation Questionnaire.

## Data Availability

The data that support the findings of this study are available from the corresponding author upon reasonable request.

## Supplementary Materials

Supplementary Materials Video S1: procedure of FSN performed by a doctor. Video S2: swaying and wrist extension movement.
